# A pilot randomised double blind controlled trial of the efficacy of purified fatty acids for the treatment of women with endometriosis-associated pain (PurFECT): study protocol

**DOI:** 10.1186/s40814-018-0274-8

**Published:** 2018-04-25

**Authors:** Ibtisam M. Abokhrais, Philippa T. K. Saunders, Fiona C. Denison, Ann Doust, Linda Williams, Andrew W. Horne

**Affiliations:** 1 MRC Centre for Reproductive Health, University of Edinburgh, Edinburgh, UK; 2 MRC Centre for Inflammation Research, University of Edinburgh, Edinburgh, UK; 3 Usher Institute of Population Health Sciences and Informatics, Edinburgh, UK; 4 MRC Centre for Reproductive Health, Queen’s Medical Research Institute, University of Edinburgh, 47 Little France Crescent, Edinburgh, EH16 4TJ UK

**Keywords:** Endometriosis, Purified fatty acids, Chronic pelvic pain, Pilot trial

## Abstract

**Background:**

Endometriosis affects 6–10% of women and is associated with debilitating pelvic pain. It costs the UK > £2.8 billion per year in loss of productivity. Endometriosis can be managed by surgical excision or medically by ovarian suppression. However, ~ 75% symptoms recur after surgery and available medical treatments have undesirable side effects and are contraceptive. Omega-3 purified fatty acids (PUFA) have been shown in animal models to reduce factors that are thought to lead to endometriosis-associated pain, have minimal side effects, and no effects on fertility. This paper presents a protocol for a two-arm, pilot parallel randomised controlled trial (RCT) which aims to inform the planning of a future multicentre trial to evaluate the efficacy of Omega-3 PUFA in the management of endometriosis-associated pain in women.

**Methods:**

The study will recruit women with endometriosis over a 12-month period in the National Health Service (NHS) Lothian, UK, and randomise them to 8 weeks of treatment with Omega-3 PUFA or comparator (olive oil). The primary objective is to assess recruitment and retention rates. The secondary objectives are to determine the effectiveness/acceptability to participants of the proposed methods of recruitment/randomisation/treatments/questionnaires, to inform the sample size calculation and to refine the research methodology for a future large randomised controlled trial. Response to treatment will be monitored by pain scores and questionnaires assessing physical and emotional function compared at baseline and 8 weeks.

**Discussion:**

We recognise that there may be potential difficulties in mounting a large randomised controlled trial for endometriosis to assess Omega-3 PUFA because they are a dietary supplement readily available over the counter and already used by women with endometriosis. We have therefore designed this pilot study to assess practical feasibility and following the ‘Initiative on Methods, Measurement, and Pain Assessment in Clinical Trials’ recommendations for the design of chronic pain trials.

**Trial registration:**

ISRCTN44202346

## Background

Endometriosis is an inflammatory condition that affects 6–10% of women in the UK [1] and is responsible for approximately 20% of gynaecological consultations. It is associated with chronic pelvic pain, pain during menstruation, pain during intercourse, and an increased likelihood of infertility [2]. Endometriosis is associated with a 45% reduction in work productivity with the annual cost for caring for women with endometriosis estimated at > £2.8 billion in the UK [1].

Endometriosis is defined by the presence of endometrial-like tissue (endometriosis ‘lesions’) outside the uterine cavity, most commonly on the pelvic peritoneum or ovaries [2]. The cause of the painful symptoms experienced by women with endometriosis is not well understood. Endometriosis is treated by surgical excision or medical management by ovarian suppression. However, up to 75% symptoms recur after surgery and medical treatments have undesirable side effects [3] and are contraceptive. There is an unmet need for new medical treatments for endometriosis.

An ideal therapy for endometriosis would (i) reduce the painful symptoms associated with the condition, (ii) reduce lesion size, (iii) preserve the patients’ ability to conceive while on medication, and (iv) have no, or limited, side effects. Oral Omega-3 purified fatty acids (PUFA) have the potential to meet all of the above criteria. Omega-3 PUFA are a Food and Drug Administration (FDA)-approved dietary supplement, which play a role in the regulation of prostaglandin metabolism and cytokine physiology, and are thought to work by competing with Omega-6 PUFA to produce lipid mediators which are anti-inflammatory as opposed to pro-inflammatory [4]. Intake of food with a high content of Omega-3 PUFA have been used in other inflammatory conditions, such as atherosclerosis, asthma, and rheumatoid arthritis and have been shown to have an anti-inflammatory effect [5].

In endometriosis, Omega-3 PUFA supplementation has been shown to reduce lesion size and reduce local prostaglandin/cytokine production in a rat model [6]. In an endometriosis model using Fat-1 mice, in which Omega-6 can be converted to Omega-3 PUFA, the number and weight of endometriotic lesions 2 weeks after inoculation were significantly less than in Fat-1 mice compared to controls who cannot convert Omega-6 to Omega-3 PUFA [7]. In vitro studies, using endometrium from women with and without endometriosis have shown that Omega-3 PUFA might have a suppressive effect on endometrial cell survival [8]. In a large, prospective cohort study investigating dietary exposure and endometriosis risk in women, it was concluded that increasing Omega-3 PUFA intake might be the first identified modifiable risk factor for endometriosis [8]. Finally, it is reported that a low ratio of Omega-3 to Omega-6 PUFA intake is correlated with painful menstruation and a high rate of autoimmune and endocrine disorders in women with endometriosis [9].

We believe that it is important to determine the true value of Omega-3 PUFA for the control of or potential elimination of endometriosis-associated pain. A definitive evaluation of the efficacy of Omega-3 PUFA in the management of endometriosis-associated pain will require a large, multicentre randomised controlled trial. This protocol outlines a pilot study to assess the processes that are unknown and vital to the delivery of such a trial, including variance of the response, variables for sample size calculation, follow-up rates, and ‘failure to comply with treatment’ rates. In the pilot study, we are using olive oil capsules, which do not contain Omega-3 PUFA, as a comparator due to lack of availability of a matched placebo. We accept that this comparator might have the potential for pain relief which could encourage participation in our trial and that it might have a unique taste with a theoretical risk of unblinding the trial medication.

## Methods/design

### Study design

**Fig. 1:**
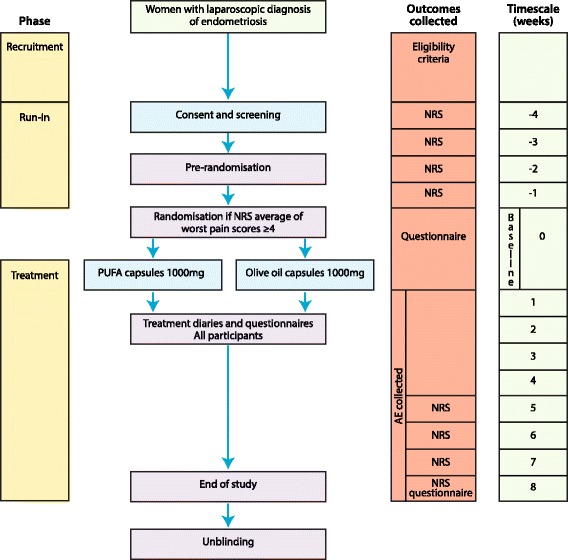
Flow chart for the trial process

We aim to perform a two-arm, parallel, double blind randomised controlled pilot trial (Fig. 1).

### Study settings

We will recruit patients from gynaecology outpatient clinics, gynaecology wards and day surgery units within NHS Lothian, UK (Royal Infirmary of Edinburgh and St. John’s Hospital).

### Study participants

Women with a surgical diagnosis of endometriosis and associated pelvic pain will be recruited.

### Primary objective

The primary objective is to assess recruitment and retention rates. Recruitment is defined as the proportion of eligible patients who agreed to participate from the number approached. Whereas retention is defined as the proportion of the participating patients who completed both baseline and follow-up visits.

### Secondary objectives

1. Estimation of variability of responses for sample size calculation
2. Acceptability of proposed methods of recruitment, randomisation, treatments and questionnaires

### Inclusion criteria

Women are eligible for inclusion if they are as follows:

- Aged between 18 and 50 years.
- Pelvic pain of > 3 months
- Pain located within the true pelvis
- Endometriosis diagnosed macroscopically by laparoscopy
- A numerical rating average ‘worst’ pain score (NRS) of greater than or equal (≥) to four (see below for details).

### Exclusion criteria

Women are excluded if they have one or more of the following:

- Unable to take/allergic to fish/PUFA/peanuts/soyabean
- Insulin-dependent diabetes
- Pregnancy
- Taking anticoagulants
- Breast feeding
- Unable to give informed consent

### Co-primary outcomes

- Recruitment and retention rates

### Secondary outcomes

- Estimation of variability of responses to inform the primary outcome of the future definitive RCT
- Acceptability of proposed methods of recruitment, randomisation, treatments, and questionnaires

### Participant enrolment

All gynaecology consultants within NHS Lothian will be sent an email informing them of the study and requesting permission to approach their patients. A clinical research fellow will approach eligible women who have agreed to be seen by the trial team, while the women are still in the clinic, provide them with patient information sheets and offer them the opportunity to discuss the trial. Consent will only be taken later once the patient has had ample time, of at least 24 h, to read the patient information sheet. A further appointment will be arranged for the participant to come for this visit.

### Screening

Eligibility for randomisation will be based on the worst of four weekly numerical rating scale (0–10; NRS) pain scores collected by text messaging (‘run-in phase’), score of 4 out of 10 in 2 or more weeks is required. Texts will be sent by the clinical research fellow to the women’s mobile phone, asking about average and worst pain, respectively, and the woman will be asked to reply to the text message with her pain score, rating it from 0 for no pain at all, to 10 being worst pain imaginable. To capture known cyclicity in pain in women, these texts will be sent once per week during the eligibility phase (weeks–minus one to minus four). If a woman has an NRS of greater than or equal to ≥ 4 in two or more of the pre-randomisation run-in weeks of the study, she will be eligible for randomisation.

### Intervention

Women will be randomised to one capsule of Omacor 1000 mgs capsules (Omega-3-acid ethyl ester filled capsules) or one capsule of comparator (olive oil soft gelatin capsules which do not contain Omega-3 PUFA) to be taken twice a day, once in the morning and once at night with food. The allocated treatments will be taken for 8 weeks. Omega-3 purified fatty acids have been used clinically, e.g. post myocardial infarction and for hypertriglyceridemia at doses of 1000–4000 mg daily (in divided doses of 1000 or 2000 mg) [10]. However, high dosage (i.e. 4000 mg) has been associated with a moderate increase in bleeding time [11], so we chose to pilot a lower dose and frequency of one capsule (1000 mg) twice a day. We approached the MHRA for advice, and due to the fact that both PUFA and olive oil are ‘food supplements’, our trial was not deemed a CTIMP.

### Randomisation

The randomisation scheme will be generated using tables, drawn up by an independent statistician, with 1:1 allocation ratio. The allocated treatment codes will be put into sealed consecutively numbered opaque envelopes. When a woman is randomised into the study, she will receive the treatment indicated in the next available envelope.

### Data collection tools

#### Pain scores

During the treatment phase of the study, pain scores will be collected once a week by text messaging during last 4 weeks of the treatment phase (weeks 5 to 8) using the same study procedures as during the ‘run-in phase’.

#### Questionnaires

Women will be given the questionnaires below to complete at baseline and 8 weeks. Baseline demographic and clinical characteristics of the participants will also be recorded at baseline.

1. Brief Pain Inventory (BPI) [12]
2. 12-Item Short-Form Health Survey (SF-12) [13]
3. Pain Catastrophizing Questionnaire (PCQ) [14]
4. Pain DETECT™ [15]
5. Sexual Activity Questionnaire (SAQ) [16]
6. Brief Fatigue Inventory (BFI) [17]
7. General Health Questionnaire-12 (GHQ-12) [18]
8. Work and Productivity Impairment Questionnaire-specific Health Problem Version 2.0 (WPAI-SHP) [19]

An additional acceptability questionnaire will be completed at the end of the study only. This questionnaire will also include questions about whether participants believed that they were receiving Omega-3 PUFA or olive oil, the acceptability of the allocated medication/treatment regimens (and compliance) and on the acceptability of completing trial-specific procedures including the questionnaires. All questionnaires will be anonymised and self-completed in private.

#### Treatment diaries

Participants will be provided with a treatment diary at the same time as their medication pack is dispensed and blood samples will be collect at baseline. All medications other than the trial treatment taken during the treatment phase of the study will be recorded in a treatment diary. This will include prescription and non-prescription treatment, such as oral analgesics, contraceptives, vitamins, topical preparations and herbal preparations.

#### Participant log

We will keep an anonymised electronic log of women who fulfil the eligibility criteria, women who are invited to participate in the study, women recruited and women who leave the trial early. Reasons for non-recruitment (e.g. non-eligibility, refusal to participate, administrative error) will also be recorded. We will attempt to collect reasons for non-participation from women who decline to take part after previously providing contact details. During the course of the study, we will attempt to document reasons for withdrawal from the study and loss to follow-up.

### Termination of study

The blinding code will only be broken in emergency situations for reasons of patient safety, where knowledge of the treatment administered is necessary for the treatment of a serious adverse event or when required by local regulatory authorities. Participants whose randomisation codes are broken will cease treatment, but will continue to be followed up. The principal investigator will document the reason for breaking the blind in the participant’s source documents and in the Investigator Site Master File. If the participant requires to be unblinded in an emergency, then the research team or pharmacy should be contacted.

### Adverse events

Participants will collect information about adverse events in their treatment diaries. They will also be instructed to contact the clinical research fellow at any time after consenting to join the trial if they have an event that requires hospitalisation or an event that results in persistent or significant disability or incapacity. Omega-3 PUFA are an FDA-approved dietary supplement, and serious adverse events are not anticipated. Any serious adverse events that occur after joining the trial will be reported in detail in the participant’s medical notes, followed up until resolution of the event and reported to the Academic and Clinical Central Office for Research & Development (ACCORD) Research Governance (http://www.accord.ed.ac.uk) and Quality Assurance Office based at the University of Edinburgh immediately, or within 24 h. ACCORD will onward report all serious adverse events (SAE) within 7 days. Hospitalisation for a pre-existing condition, e.g. exacerbation of pain due to endometriosis will not be considered a SAE, but will be documented.

### Sample size

The emphasis in this study is to establish feasibility, not statistical significance. This study is designed primarily to explore the recruitment and retention rates, and we will aim to recruit as many women as possible over a 12-month period. We estimate that we will recruit ~ 4–5 patients per month and will aim to recruit 40 patients. As we anticipate a recruitment rate of 50%, 40 participants would be able to give 95% confidence interval of 35–65%. The data from this pilot study will be used to refine sample size calculations for any future RCT.

### Data analysis

#### Recruitment and retention rates

Using the information collected from the participant log, we will determine the number of patients recruited from the pool of eligible women and a > 50% recruitment will be deemed acceptable. While a retention rate of 100% would be ideal, we will consider a rate of 80% satisfactory. We will provide an estimate of the proportion and its 95% confidence interval. In addition, we will determine the nature and number of unanswered questions in each questionnaire. We aim to determine whether the trial design will perform well enough in the field to warrant rolling out the study to full trial. All analysis will be carried out at the University of Edinburgh.

#### Effectiveness and acceptability of proposed methods of recruitment, randomisation, treatments and assessment tools

These areas will be assessed quantitatively using additional questions included in participant questionnaires administered at 8 weeks. The data collected from the different questionnaires will be used to explore comparisons between the two groups and will be reported as estimates of difference and corresponding confidence intervals in order to calculate sample size. Due to the conflicting literature about the benefits of methods such as prescription monitoring, pill counting and devices for monitoring the self-administration of medicines, data on blinding and compliance to treatment will be derived from questionnaires at 8 weeks. We aim to determine if treatment is acceptable in terms of self-reported compliance (from treatment diaries). We will examine the effect of non-compliance and retention, which will be used to inform the sample size required for a full trial.

### Trial sponsors

The trial is co-sponsored by the University of Edinburgh and NHS Lothian.

### Data handling, storage and archiving

A log with the patients’ name and date of birth will be kept along with their unique study number in a separate file. All the data generated from the study will be stored in an anonymised form in a bespoke database, which will also be password protected. Only anonymised information will be stored on this, and participants will only be identifiable by their study number. All paperwork will be kept in a locked filing cabinet in a locked office. All the data will be stored on university server on a password-protected computer with a limited access to the research team, in accordance with the NHS and University of Edinburgh guidelines and in accordance with the Data Protection Act. All study documentation will be kept for a minimum of 5 years from the protocol defined end of study point. When the minimum retention period has elapsed, study documentation will not be destroyed without permission from the sponsor. The trial statistician will also be blinded to the treatment during analysis.

### Ethics

Ethical approval has been obtained from the Scotland A Research Ethics Committee (LREC 16/SS/0010).

### Dissemination

The data will be presented at international conferences and published in peer-reviewed journals. The clinical study report will be used for publication and presentation at scientific meetings. We will make the information obtained from the study available to the public through national bodies, e.g. Endometriosis UK.

## Discussion

We believe that a definitive evaluation of the efficacy of Omega-3 PUFA in the management of endometriosis requires a multicentre randomised controlled trial. Recognising that there may be potential difficulties in mounting a large randomised controlled trial for the management of endometriosis-associated pain using a supplement which is widely available over the counter, we have designed this pilot study to assess practical feasibility following the Initiative on Methods, Measurement, and Pain Assessment in Clinical Trials (IMMPACT) recommendations for the design of chronic pain clinical trials [20]. We plan to randomise participants to either Omega-3 PUFA or olive oil and then evaluate recruitment and retention rates as our primary outcome.

A potential limitation of our study is the broad inclusion and exclusion criteria which we have produced in an attempt to reflect the true clinical scenario of endometriosis. Our criteria do not take into account pain intensity, do not exclude women with non-reproductive comorbidities (e.g. irritable bowel syndrome, bladder pain syndrome) that could explain their symptoms and allow participants the use of concomitant medications. We are aware that these characteristics may increase variability in patient responsiveness to treatment and carry the risk of failing to demonstrate treatment effect. We will therefore capture this information in our pilot study in the participants’ questionnaires and treatment diaries to inform interpretation of our results and the planning of the future randomised controlled trial. Like many of the medications/supplements used for endometriosis, Omega-3 PUFA given in the highest effective dose with the rate and severity of adverse effects are minimised.

One of the other limitations of our pilot study is the lack of availability of a suitable placebo. It was not possible to find a placebo identical to Omega-3 PUFA, and therefore, we decided to use a ‘comparator’ (olive oil) instead of a true placebo. We thought that olive oil, a comparator with the potential for pain relief [21], was the best option for our target population to encourage participation in our trial [22]. The drawback of using olive oil is that it may have a unique taste and there is a theoretical risk of the subjects guessing which arm of the trial they have been randomised to. In order to determine whether significant ‘unblinding’ was present, we will ask the subjects at the conclusion of the trial to guess what treatment was received and the primary reason for the guess in an attempt to determine the validity of using olive oil as a suitable comparator. Another limitation of our study is the short timescale of the treatment phase, and we are aware that a longer trial will likely be necessary to better assess the long-term effects of Omega-3 PUFA.

We have chosen data collection tools to assess the core domains of pain, physical/emotional functioning (including sleeping difficulties), improvement/satisfaction with treatment, symptoms and adverse events, following advice from a clinical psychologist and a general practitioner, both with a research interest in endometriosis. These tools are in line with the IMMPACT recommendations for chronic pain trials [23]. We will assess the reliability and acceptability of these data collection tools in order to refine our methods for the subsequent RCT.

In summary, this pilot trial with embedded evaluation of trial processes and collection of outcome data will allow us to undertake detailed feasibility work to inform a future large-scale trial in the important but challenging area of dietary supplements and endometriosis-associated pain.

## Abbreviations

AA: Arachidonic acid
ACCORD: Academic and Clinical Central Office for Research and Development
BFI: Brief Fatigue Inventory
BPI: Brief Pain Inventory
CRF: Case report form
DHA: Docosahexaenoic acid
EPA: Eicosapentaenoic acid
FDA: Food and Drug Administration
GHQ-12: General Health Questionnaire-12
IMMPACT: Initiative on Methods, Measurement, and Pain Assessment in Clinical Trials
NRS: Numerical Rating Pain Score
PCQ: Pain Catastrophizing Questionnaire
PUFA: Purified fatty acids
RCT: Randomised controlled trial
SAQ: Sexual Activity Questionnaire
SF-12: 12-Item Short-Form Health Survey
WPAI-SHP: Work and Productivity Impairment Questionnaire-specific Health Problem Version 2.0
